# Early development of local data dashboards to depict the substance use care cascade for youth involved in the legal system: qualitative findings from end users

**DOI:** 10.1186/s12913-024-11126-5

**Published:** 2024-05-30

**Authors:** Allyson L. Dir, Lauren O’Reilly, Casey Pederson, Katherine Schwartz, Steven A. Brown, Khairi Reda, Logan Gillenwater, Sami Gharbi, Sarah E. Wiehe, Zachary W. Adams, Leslie A. Hulvershorn, Tamika C.B. Zapolski, Malaz Boustani, Matthew C. Aalsma

**Affiliations:** 1 Department of Psychiatry, Indiana University School of Medicine, 410 W. 10th St. Suite 2000, Indianapolis, IN 46222 USA; 2 Department of Pediatrics, Indiana University School of Medicine, Indianapolis, IN USA; 3 Department of Biostatistics and Health Data Science, Indiana University School of Medicine, Indianapolis, IN USA; 4 Luddy School of Informatics, Computing, and Engineering, Indiana University-Indianapolis, Indianapolis, IN USA; 5 Department of Medicine, Indiana University School of Medicine, Indianapolis, IN USA

**Keywords:** Data dashboards, Behavioral health services cascade of care, Youth involved in the legal system, End-user feedback

## Abstract

**Introduction:**

Rates of substance use are high among youth involved in the legal system (YILS); however, YILS are less likely to initiate and complete substance use treatment compared to their non legally-involved peers. There are multiple steps involved in connecting youth to needed services, from screening and referral within the juvenile legal system to treatment initiation and completion within the behavioral health system. Understanding potential gaps in the care continuum requires data and decision-making from these two systems. The current study reports on the development of data dashboards that integrate these systems’ data to help guide decisions to improve substance use screening and treatment for YILS, focusing on end-user feedback regarding dashboard utility.

**Methods:**

Three focus groups were conducted with *n* = 21 end-users from juvenile legal systems and community mental health centers in front-line positions and in decision-making roles across 8 counties to gather feedback on an early version of the data dashboards; dashboards were then modified based on feedback.

**Results:**

Qualitative analysis revealed topics related to (1) important aesthetic features of the dashboard, (2) user features such as filtering options and benchmarking to compare local data with other counties, and (3) the centrality of consistent terminology for data dashboard elements. Results also revealed the use of dashboards to facilitate collaboration between legal and behavioral health systems.

**Conclusions:**

Feedback from end-users highlight important design elements and dashboard utility as well as the challenges of working with cross-system and cross-jurisdiction data.

**Supplementary Information:**

The online version contains supplementary material available at 10.1186/s12913-024-11126-5.

## Highlights

End-user feedback on data dashboards improved its usability and acceptability.

Cross-system data harmonization was a primary challenge to dashboard development.

Dashboards with multi-system data facilitated cross-system collaboration and decision-making.

**Supplementary Information:**

The online version contains supplementary material available at 10.1186/s12913-024-11126-5.

## Background

Youth involved in the legal system (YILS) have high rates of substance use compared to their non legal system-involved peers [1]; however, they are also less likely to receive treatment for substance use [2]. Ensuring YILS are connected to substance use treatment is critical, especially as those who use substances are more likely to remain involved in the legal system into adulthood [3]. The Juvenile Justice Behavioral Health Services Cascade (hereafter, “Cascade”) was developed as a tool to illustrate and quantify the process of identifying YILS in need of substance use treatment and connecting them to behavioral health services in the community [4]. The Cascade defines individual steps along a successful care continuum, which require navigation across both the juvenile legal system (JLS) and the behavioral healthcare system (here, local community mental health centers [CMHCs]). Steps include: (1) identification of youth need for substance use treatment through screening in the JLS, (2) referral to behavioral health services at local CMHCs, (3) behavioral health service initiation, and (4) service engagement [4]. Examining Cascade trends (e.g., rates of completion of different Cascade steps) in a jurisdiction can help identify gaps in the care continuum and ideally guide needed improvements to policies and processes that impact Cascade step achievement (e.g., improving JLS referral processes to improve referral and treatment initiation step rates).

The current manuscript describes our team’s *development* of data dashboards of the Cascade for use by decision-makers within CMHC and JLS (i.e., the end users). This work was conducted as a part of a larger study, “ADAPT” (Alliances to Disseminate Addiction Prevention and Treatment: A statewide learning health system to reduce substance use among justice-involved youth in rural communities; UG1DA050070), a NIDA-funded research project conducted among eight Indiana counties with the goal of improving YILS Cascade outcomes through collaboration across local JLS and CMHCs [5]. ADAPT utilizes a Learning Health System approach to establish cross-system alliances and equip alliances with data to identify localized solutions to address gaps in the Cascade. Data dashboards depicting the Cascade were implemented as one component of ADAPT, updated regularly with data linked and integrated from the two systems, and made available to alliance teams (i.e., JLS probation and CMHC staff) to facilitate data-driven decision-making to improve youth outcomes. Prior to ADAPT, there were no formal processes in place for JLS and CMHCs to share data in these jurisdictions, nor did structured alliances between CMHC and JLS exist in these jurisdictions.

Data dashboards have become ubiquitous in recent years with applications in a wide range of domains, including business intelligence [6], healthcare [7, 8], and public health [9, 10]. A foundational principle in dashboard design is to provide visual summaries of key data and metrics so they can be interpreted quickly and with little effort [11]. Effective presentation of pertinent data can, in turn, support a variety of information processing tasks, such as monitoring reporting and decision-making [12, 13]. In the case of ADAPT, we proposed to develop dashboards as a tool for JLS and CMHC alliances to identify gaps in the Cascade in their jurisdiction and support decision-making regarding needed programs or system-level changes that would address identified Cascade gaps. Given the complex nature of administrative data from these two systems, dashboards offer an ideal solution to summarizing and visualizing such data.

Beyond improving single-user information access, dashboards can promote collaborative decision-making between individuals, departments, or across agencies [14, 15]. By offering a shared representation that is updated with real-time or near real-time data, dashboards enable individuals to engage in “data conversations” [16], thus advancing the analytic and communicative needs of collaborative organizations [17]. This is particularly ideal in the case of facilitating collaboration between JLS and CMHC systems, whose unique policies and procedures can make collaboration challenging [18]. Visualization of data from both systems allows for opportunities for education and better understanding of each system’s procedures.

The current study is situated within the *process* of developing dashboards for local JLS and CMHC alliances, which entailed approaching dashboard development from a user-centered perspective. Many dashboards remain underutilized due to developers’ misunderstanding of end-user data and analytic needs and employing ineffective visualization techniques [11, 19]. As such, end-user focus groups and iterative development is a fundamental part of the design process; thus we highlight that process here. The current manuscript aims to (1) present end-user feedback regarding early dashboard design, (2) illustrate how feedback was integrated into the dashboard design, and (3) discuss challenges to dashboard development and potential dashboard uses.

## Methods

### Dashboard Development

Dashboard development was conducted prior to focus groups. Below we discuss the initial process of dashboard “mock-ups” that were developed for focus groups.

### Data harmonization

We consulted with JLS and CMHC representatives from each of the counties who were familiar with data systems to ensure appropriate understanding of the data from their systems and determine appropriate data points to best define cascade steps. The primary data sources utilized to develop the dashboard were JLS and CMHC administrative data. In the participating JLS jurisdictions, one of two established data management systems are commonly used to capture legal case records of YILS: Quest Case Management System (Quest) or the Summary Reporting System (SRS). These two systems differ in the ways they structure, organize, and manage data. Behavioral health service utilization data were also collected from local CMHCs participating in ADAPT, again transferred to the university from various electronic health record systems. Behavioral health utilization data included variables derived from the CMHCs’ billing records, which included number of outpatient visits to a CMHC billed per youth post arrest. Individual procedure codes allowed us to distinguish outpatient visits from inpatient or medication-management services but were not classified further for display on the dashboard. To track YILS completion of Cascade steps (specifically behavioral healthcare initiation), YILS data were record-linked to CMHC records at an individual level using youth identifying information including first, middle, and last name, date of birth, social security number, gender, race, ethnicity, and residential address including zip code. We performed the linkage by using 20 deterministic matching algorithms and 5 probabilistic matching algorithms. True matches were identified by the deterministic algorithms. A conservative matching score threshold was selected for each of the probabilistic matches, above which true matches were selected. For each probabilistic algorithm, a manual review of each match under the threshold score was performed to hand pick any remaining true matches. A final round linking all true matches was performed to establish a unique ID per youth participant.

Because of the disparate data management systems employed by participating agencies, data gathered to populate the dashboards were first harmonized across systems to ensure consistent variable- and value-naming conventions and similarity in concepts recorded. This was accomplished by first creating an internal common data model for both the JLS and CMHC data sources and then mapping each data source to the applicable model. Within this process, some free-text fields from each data source were manually reviewed and used to create jurisdiction-specific definitions for Cascade step completion and other analysis variables of interest. As one example of data harmonization, in some JLS systems, juvenile probation officers were responsible for completing referrals to CMHC services, while in other systems referrals were entered by courts; since both system procedures denote “referrals” in the cascade, these unique data points were both considered referral. In addition, to track YILS completion of Cascade steps (specifically behavioral healthcare initiation), YILS data were record-linked to CMHC records at an individual level using youth identifying information including first, middle, and last name, date of birth, social security number, gender, race, ethnicity, and residential address including zip code. Matching based on deterministic algorithms was identical to the approach described above. True matches were then assigned a unique ID to represent one ID per youth participant. Once data were harmonized and linked, author SB created a dataset capturing YILS participants and their achievement of each Cascade step along the continuum of care. Tableau data visualization software (version 2022.1) was used to read in the dataset and develop the data dashboard. Following initial development, we planned for data updates within Tableau on a quarterly basis.

### Data visualization

Once data were harmonized and linked, author SB created a dataset capturing YILS participants and their achievement of each Cascade step along the continuum of care. Tableau data visualization software (version 2022.1) was used to read in the dataset and develop the data dashboard. Following initial development, we planned for data updates within Tableau on a quarterly basis. Initial mock-ups of the dashboard and many individual data visualizations were originally developed through ongoing discussions between the university-based research team and data analysts from a local firm, Empact Solutions, which provided expertise and experience visualizing local JLS data. The dashboard views were developed in an iterative manner as the team met weekly to discuss potential designs and uses for each new visualization. Example topics of consideration included: sample inclusion criteria per visualization, interactive filters needed to review data regarding specific subgroups of YILS, and approaches to limit reidentification of YILS. Once created, the dashboard mockup was then transferred back to the university research team and shared with ADAPT participants through the university’s secure web-based Tableau server environment.

Dashboard mockups were then utilized for focus groups which we discuss below. To note, following focus groups, the data team then conducted necessary modifications based on focus group feedback. Additional technical details on data manipulation and visualization required to address recommended changes are not presented in the current manuscript, although in the results we do present images of visualizations pre and post focus group feedback.

### Procedures

We recruited data dashboard end users from JLS and CMHCs in eight different counties participating in the larger parent study, ADAPT. We recruited individuals interacting who would be end-users, including individuals directly with youth on the front lines (i.e., juvenile probation officers and CMHC service providers), as well as those in roles of decision-making regarding system-level processes (i.e., judges, probation supervisors, and CMHC supervisors).

Three focus groups with JLS and CMHC personnel were conducted with *N* = 21 participants total representing 8 counties (focus group 1: *n* = 7 JLS, *n* = 2 CMHC, 5 counties; focus group 2: *n* = 3 JLS, *n* = 2 CMHC, 4 counties; focus group 3 *n* = 4 JLS, *n* = 3 CMHC, 3 counties). Focus groups took approximately one hour. The goal of the focus groups was to pilot the dashboards and collect end-user feedback regarding the acceptability and usability of the dashboard and suggestions for needed modifications to improve usability. Across the three focus groups we utilized a semi-structured interview guide with questions asking about perceived utility of the dashboard, such as using as a means of identifying cascade gaps as well as their interpretation of the data as presented in the initial dashboard mock-ups (with the intention of capturing needed modifications to how data were presented). Also, given that dashboards were a novel tool to both CMHCs and JLS, and further, given that data sharing was not a common practice prior to ADAPT, participants were also educated on the cascade steps and provided suggestions on how the dashboard could be utilized. Specifically, we discussed the potential use of the dashboard as a way to not only monitor the service cascade but also to identify gaps in the service cascade and potentially use data as a measure to test potential solutions to service gaps (e.g., using data to measure success of changes to a referral process between JLS and CMHCs). In each of the focus groups, a research team member guided individuals through different dashboard views and functions. Focus groups were then audio transcribed and coded. Following completion of the focus groups, feedback was reviewed by the research team and modifications were made to the dashboard accordingly. All study procedures were approved by the Indiana University IRB.

### Analyses

Transcribed focus groups were uploaded to NVivo for coding and analysis. We conducted a descriptive analysis to summarize topics on how JLS and CMHC relate to data based on their own work experiences [20, 21]. First and second authors developed qualitative topics inductively. Initial coding by the first author was conducted to develop a set of initial codes. Next, first and second authors conducted additional coding with a subset of transcripts to further refine the initial set of codes, as well as additional codes that, together, were considered the final set of codes. Finally, focused coding with all transcripts was conducted by the first and second author; coders met frequently throughout to resolve any disagreements in coding and ensure 100% reliability.

## Results

Across the three focus groups, topics emerged regarding suggestions for dashboard modifications to improve usability. Below we discuss these topics along with images of dashboard views illustrating changes made based on feedback.

### Dashboard visualization and aesthetics

#### Improving aesthetics, color, and interactive features

**Fig. 1:**
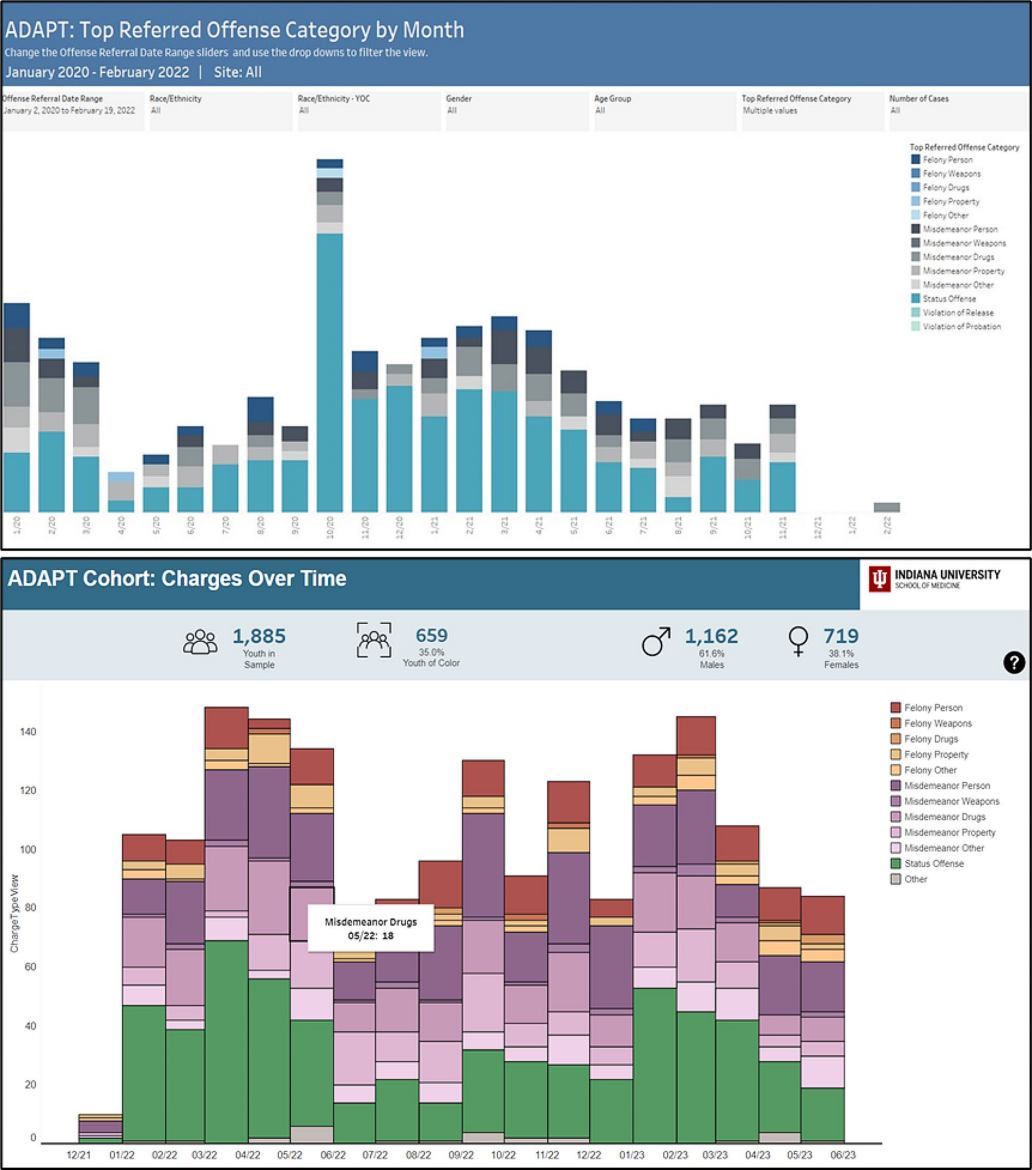
Displays changes made to better utilize color schemes. Top: Draft dashboard version with little color contrast; participants noted colors were difficult to differentiate. Bottom: Updated dashboard with different colors and clear color scheme. Colors were also used to organize categories of data – in this view colors organize types of offenses: felonies, misdemeanors, and status offences

Focus group participants offered several suggestions for improving dashboard aesthetics and data interpretability. A key recommendation was to employ colors more purposefully for organizing and imparting meaning onto the displayed data categories. While the initial dashboard predominantly used color for visual appeal (as illustrated in Fig. 1), many participants looked to use colors for the semantic grouping. For example, one participant suggested colors “gradations” to convey different levels of treatment completion: “set the color scheme up to indicate how far a kid gets in the process.” Participants also stressed the need for consistent and distinctive color mapping throughout the dashboard. For example:

Whatever your color scheme is here, match that to the other color scheme, where if blue means something happened that was supposed to happen, here make blue the thing that this was what was supposed to happen in earlier graphs. Make blue always the good thing that happened and orange the thing that was bad… So have that consistency so it’s easier to then read this all the way through.

To accommodate this feedback, we adopted a modified color scheme that maximizes the perceptual discriminability and “nameability” of colors associated with different categories. For example, we opted to represent different offense categories with distinctive hues (red, orange, and yellow for various felony classes, purple for misdemeanors, and green for lesser offenses). Within each category (e.g., misdemeanors), we varied lightness to show gradations of offenses (e.g., weapons- versus substance-related misdemeanors in dark and light purple, respectively). Figure 1 shows screenshots of the original and finalized color scheme based on participants’ feedback.

**Fig. 2:**
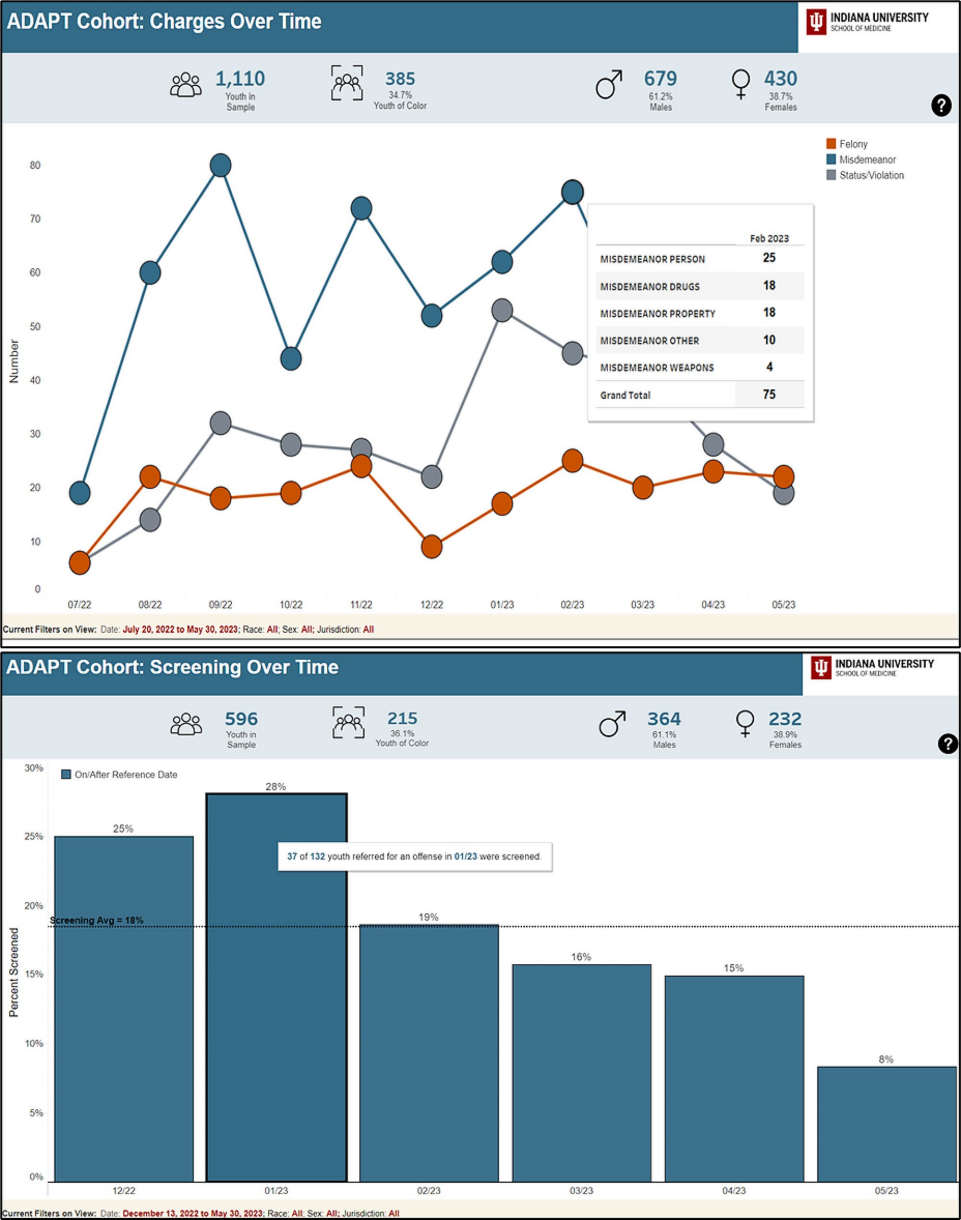
Top: Demonstrates hovering options that were added to provide data details. Bottom: Demonstrates trend lines and “benchmarking” that was added for reference

In addition to utilizing colors for meaning and clarity, participants also offered suggestions for more clearly labeling data and providing numbers represented in the charts. For example, one participant offered: “Are there ways to get any numbers in any of those categories? I mean, obviously, you can look at it and go ‘Oh, that pretty color is a lot’ compared to how many, were there a thousand referrals or 20?” Another noted: “It would be even more useful if when you hovered over it, it would say violation of probation and the number of those or something like that…I can use that as my way of seeing what all is included in each of these bar graphs.” As a result, data labels were added to the dashboard and hovering functions were added to improve ease of interpretation. Figure 2 illustrates many of the functions that were added to the dashboard based on focus group suggestions.

#### Improving dashboard element definitions

**Fig. 3:**
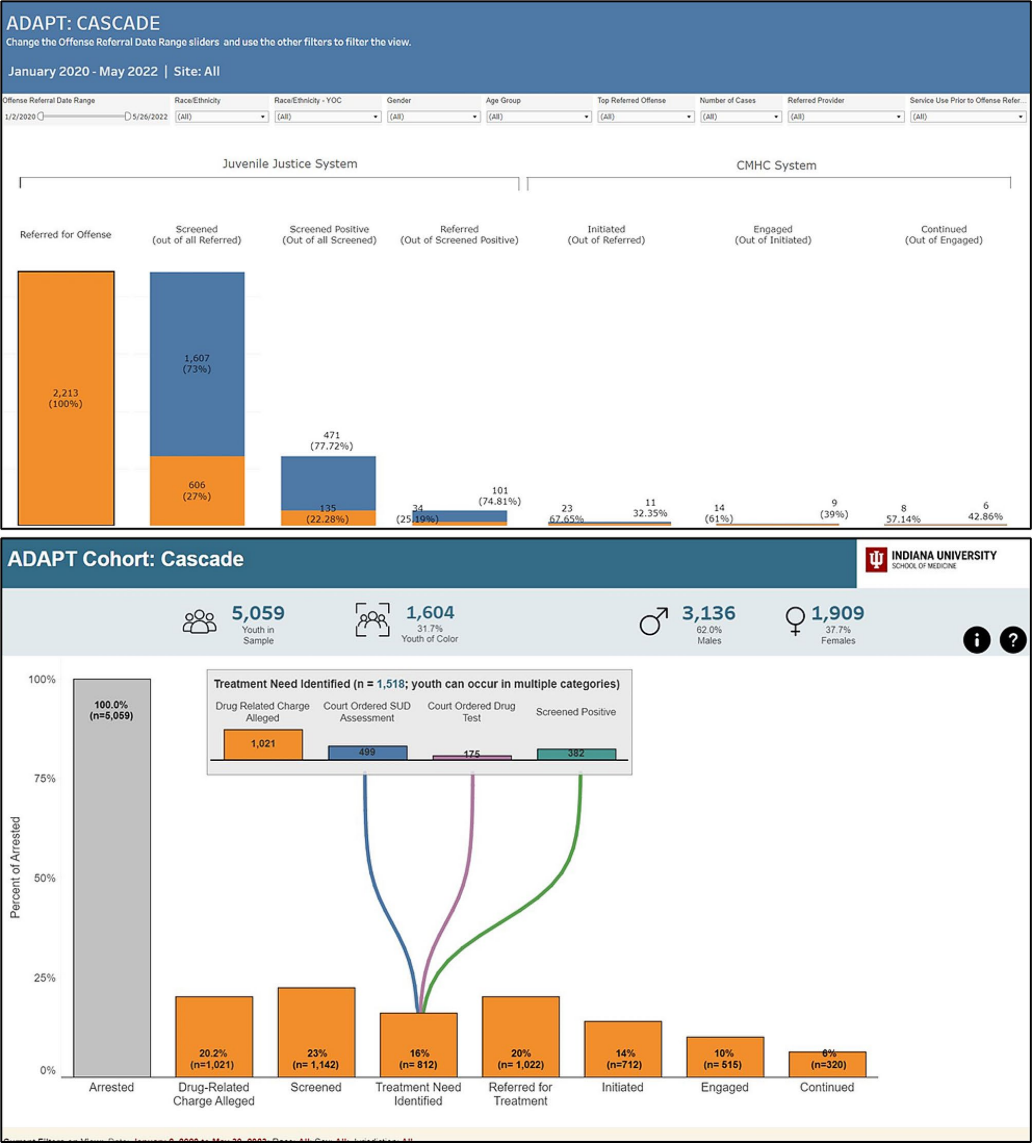
Images show earlier (top) and modified (bottom) iterations of the Cascade. Top: In early versions of the cascade, bars represented the percentage who completed each step in reference to those who completed the previous step (i.e., denominator changed for each step based on who completed previous step). Bottom: Modified cascade view showing only percentage of youth completing each step in reference to the entire sample of youth arrested. Due to data entry differences across jurisdictions and differences in jurisdiction procedures, not all individuals had the opportunity to complete all Cascade steps. Thus, we eliminated illustrating the denominator as those who completed the previous step. Additional data were also added to identify alternative ways that individuals were identified as needing treatment based on different county JLS procedures

One of the challenges that individuals noted in understanding the data as displayed on the dashboard was regarding clarity on local definitions of the Cascade steps. For the “screened” step (i.e., who received a screen for substance use risk), participants suggested providing information on the screening instrument used since this varies across jurisdictions. For example, as one JLS personnel noted: “[Does] screened mean when we would initiate the CRAFFT [i.e., a six item self-report measure]? Because then the next one says, ‘screened positive.’ Screened positive for a urine screen or the CRAFFT?” Another example of where clarification was needed related to the use of the word “referred” within the dashboard. JLS terminology uses “referred” to mean the time at which a youth becomes involved in the legal system (e.g., youth is arrested or otherwise recommended to go through probation intake). In contrast, “referred” as step within the Cascade indicates whether JLS staff recommended youth for treatment. One CMHC participant noted: “The referred section, it was a little confusing, especially for community people that are looking at this. So, I think we requested that they would change it to “refer to CMHC,” which then makes it a little less confusing when you have referred on both columns.” Participants also highlighted challenges in understanding how constructs were defined within partnering agencies (i.e., JLS understanding CMHC data points and vice versa) and across jurisdictions. For example, as one CMHC participant noted: “I don’t know enough about types of offenses…, but I suspect that, that’s a lot more helpful for the juvenile justice partners on the call. I come from mental health. I’m not quite sure what all those mean, but I think they’re going to be highly meaningful to others.” Similarly, clarification was also suggested for the cascade step of “engaged” (i.e., engaged in treatment), as one JLS participant noted: “First thing that really catches me - and I can’t seem to get away from - is ‘engaged,’ to me that’s a matter of perspective and so I wonder, what criteria does that cover? Is that ‘attends regularly,’ because that’s a matter of perspective that word throws me a little bit.” These suggestions resulted in several modifications made to the Cascade views, as we present in Fig. 3. Though not pictured, the repeated conversations about data element definitions also led to development of a dashboard glossary and FAQ section accessible with a single click on each dashboard view.

### Improving dashboard utilization

#### Benchmarking and facilitating data comparisons

Across all focus groups, participants emphasized the desire to use the dashboard as a means of benchmarking between and within sites. Many participants were interested in being able to compare data and metrics in their county with other counties or compare metrics over time. For example, as one JLS participant noted: “If you’re looking at this from a county level, maybe you want to see your county compared to all of them. And maybe then you can make an argument for how fast your court system works versus others or something like that.”

A CMHC participant also noted the need for benchmarking:

Because I oversee multiple locations, it would be helpful…to see kind of the average across the board and compare that to what my local county averages are on some of these, so that way we can kind of identify does it seem like we’re doing better in some areas or we’re really struggling in some areas compared to everyone…I think that’s helpful information if we’re looking to improve services in our county.

**Fig. 4:**
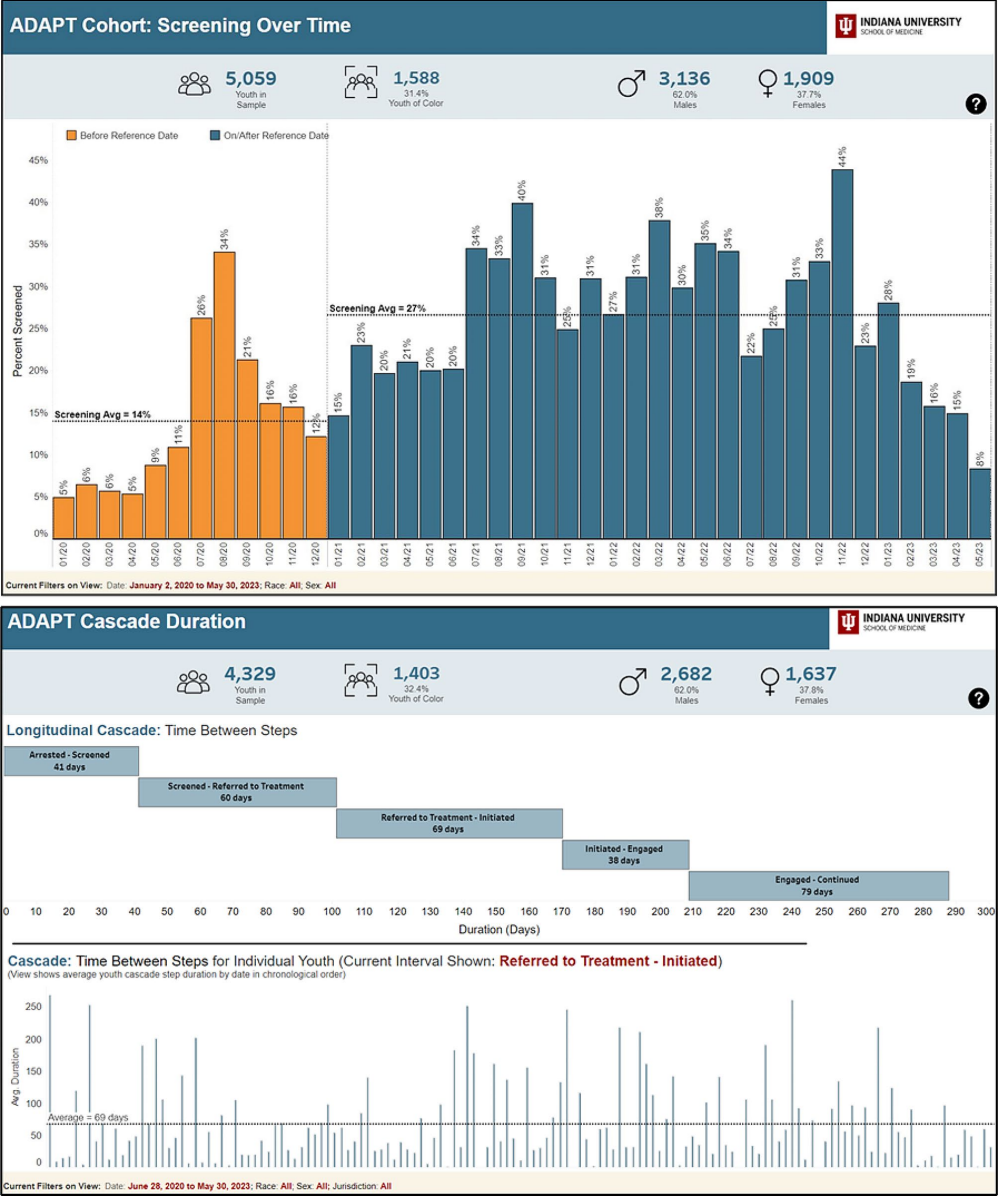
Updated dashboard views illustrating benchmarking and filtering options. Top: Benchmarking is used here to compare rates of SU screening across two time frames (before and after implementation of substance use screening). Trend lines illustrate average across time frame and colors are utilized to differentiate time frames. Bottom: Image demonstrates filtering options which were added to be able to compare different groups or examine more specific data. The top part of the image illustrates the average time between each step across the sample. In the bottom half, the step “referred to treatment – initiated” was chosen to filter out and examine the distribution of all youth in the sample with respect to time between this specified step

Participants also wanted the ability to make comparisons within their own county, such as being able to compare data and metrics across different time periods or make comparisons among different groups; participants suggested adding trend lines or other functions to easily compare averages to improve the usability. For example, one participant from JJ suggested: “If you could add like a trend line on there somehow or… add something that allows you to narrow down specific dates. So maybe I don’t want to see every month, maybe I want to see like a six month period or something…” Others also requested trend lines in order to more easily identify trends in the data: “You can kind of see the trends, but sometimes having those trend lines there or like the comparison lines or average trends or something comparing the two lines, sometimes that’s kind of a helpful extra point of reference.” As a result, benchmarking, averages, and trend lines were added to the dashboard as illustrated in Fig. 4.

#### End-user dashboard feedback as a facilitator of collaboration

Across focus groups, conversation about the dashboard naturally facilitated collaborative cross-system and cross-jurisdiction education. For example, in one focus group, JLS personnel across four different counties engaged in a discussion of their processes for substance use screening, which offered a rare opportunity to make comparisons and inform partners of differences. “A lot of departments, including my own, we don’t hear what other departments are using for screening tools and finding helpful…And I would be curious to know what other departments are using that they’re finding helpful.” Similarly, dashboard demonstration also fostered conversations between JLS and CMHC personnel and provided and opportunity for these two systems to educate one another and collaborate. For example, in one focus group both CMHC and JJ staff were reacting to data presented regarding the time from initial arrest to treatment initiation (as illustrated by the Cascade depicted on the dashboard) and this prompted an informative discussion. One CMHC provider started the conversation:

I think we all have issues with like backlog and stuff…I can say at a CMHC, we’re probably depending on payer. There’s certainly a couple weeks delay potentially. Again, if we’re getting enough of a referral base to justify kind of creating more space for those things, I think we could probably come up with some solutions.

JLS responded:

From our side, from the arrested to screened…we have one intake officer who does most of those screens when she does their preliminary inquiry. And sometimes we get 20 cases referred in a week so we do get backlogged pretty far. Sometimes four weeks, six weeks out, depending on how many referrals we get. So I can definitely see how we may be stretching that out to that 109 days.

This conversation illustrates the utility of the dashboard in facilitating such cross-system collaboration and a potential process for problem-solving.

## Discussion

The aim of the current study was to summarize qualitative results from three focus groups that provided feedback on the development of data dashboards depicting local views of the Cascade for YILS, as well as provide visualizations of the updated dashboards after feedback integration. End users were those working in the JLS (e.g., probation officers) or CMHCs (e.g., mental health counselors). Qualitative analysis from the focus groups identified several key topics, including (1) the importance of aesthetic and functional elements of the dashboard, (2) the need for clear definitions of data constructs, and (3) the potential utility of the dashboard as a tool to facilitate collaboration between JLS and CMHCs.

First, end users provided valuable feedback regarding dashboard aesthetics and functional components that were incorporated to improve dashboard utility. End users were drawn to use of color to help organize and increase understanding of data [19, 22]. Use of colors has been shown to facilitate better understanding of data among dashboard users by evoking semantic associations [23]. Filtering and benchmarking functions were also added to the dashboard to accommodate specific tasks requested by JLS and CMHC end users. For example, participants wanted the ability to examine specific data points or subgroups of individuals, such as examining only felony cases or examining cases according to race. As such, options to filter data according to specific characteristics were added. Additionally, participants from both the JLS and CMHCs explained the importance of being able to identify trends or averages over time or across different jurisdictions and being able to compare current data with these trends. For example, participants from the JLS were interested in comparing rates of substance use screening in different jurisdictions, while CMHC users were interested in examining trends in service use over time.

Second, operational definitions of cascade variables were paramount to dashboard development and we encountered many challenges in developing definitions that were consistent with the available data and relevant and useful to end users. There were several practical considerations that made defining and disseminating clear data definitions for end users challenging. Dashboard development required included linking data from the JLS and CMHCs across eight diverse counties, introducing considerable complexity and variation in data harmonization processes and challenges with both JLS and CMHC data. Below we discuss challenges with both JLS and CMHC data.

JLS processes, data entry practices, and data definitions vary by county; therefore, data harmonization to construct dashboards for eight different counties required considerable ongoing data management effort and ongoing input from JLS end users in each county. For example, the timing of data capture among probation departments differed widely, which had significant implications for the sample of youth reflected within data dashboards. Notably, in some jurisdictions, YILS cases were only entered into the local data management system once the juvenile prosecutor decided to pursue legal intervention, resulting in smaller and more narrow samples of youth. In other locations, youth were entered into the system as soon as an arrest was logged, resulting in samples with more youth and a wider range of youth characteristics, offense types, and outcomes. As such, this created significant challenges in end users being able to compare their own county’s data with other counties, which was identified as an important function for end users.

Challenges were also seen with cascade step definitions involving CMHC data (treatment initiation, treatment engagement, treatment completion). Like JLS, CMHC processes, medical record systems, and data entry vary across agencies. Even though definitions used as a part of this study were based on seminal research in the area [24], there was disagreement among end users in the utility of such definitions and relevance to their own processes. For example, “treatment engagement” was defined as engagement in services for six weeks; however, many CMHC representatives noted that this definition was not representative of many of their service and program metrics for engagement. This is particularly relevant in the case of substance use treatment; there is a continuum of services for substance use with varying intensity and duration, and thus, level and type of service may require unique definitions of engagement and treatment completion [25, 26]. Despite unique considerations across jurisdictions, end users noted the importance of being able to compare their county data with other counties; thus, extensive efforts were made to ensure that data represented the jurisdiction but could also be compared with other jurisdictions.

Taken together, end users played a critical role in dashboard development and findings underscore the importance of using a user-centered design approach when developing data dashboards. User-centered design refers to an approach to innovation development (e.g., data dashboard) that intentionally and continuously incorporates end user feedback into the development process in order to maximize utility and shorten the learning curve [27–29]. Many dashboards remain underutilized due to developers’ misunderstanding of end-user data and analytic needs and employing ineffective visualization techniques [11, 19]; thus, incorporating end user feedback through user-centered design ensures that components necessary to optimize functionality are included (e.g., being able to compare with other counties) and help make the final product more acceptable to end users. In the current study, this was paramount given that dashboards were intended for end users in two different systems with differing priorities, and further, that dashboards needed to be tailored to different counties.

Although the primary purpose of the focus groups was to get feedback on initial dashboard iterations, they also revealed the potential utility of dashboards in facilitating cross-system collaboration. During all of the focus groups, conversation organically turned into discussion among JLS and CMHC participants; discussions involved CMHC and JLS individuals educating one another about system processes and explaining data trends within their system as well as brainstorming how they could improve cascade outcomes.

This collaboration was also promising given this was one of the larger goals of the parent study – to increase cross-system collaborative decision-making. This is consistent with literature on dashboard’s utility in facilitating problem-solving discussions [10]. Moreover, in the case of the Cascade, such problem-solving requires collaboration between two large systems – JLS and CMHC – with unique processes and procedures which can make collaboration challenging. Results from these focus groups offer a glimpse into the potential utility of the dashboards to facilitate the cross-system collaboration needed for local decision-making. The dashboard served as a central source of collective sensemaking for pertinent stakeholders regarding where youth are being lost along the care cascade. We anticipate that this shared understanding of an issue will facilitate decision-makers to coordinate their efforts in improving different steps of the cascade. While dashboards may not directly impact cascade steps, the goal is for the dashboards to illuminate gaps in the cascade (e.g., poor retention in services) and provide an opportunity for collaborative decision-making on solutions to improve cascade steps. This is particularly relevant for substance use; individuals with substance use disorders are often involved in multiple systems (e.g., criminal justice, child welfare, health systems); use of data dashboards may be helpful in improving treatment retention. The overall goal of dashboard development as part of ADAPT is to use dashboards as a tool in learning health system alliances (which were implemented as part of ADAPT). Future research will examine the actual utilization of the dashboards in the alliances (i.e., frequency of use by data element such as number of days between visits and measures of disparity, quantify which data elements were used to guide interventions, etc.). Dashboards will also be utilized to measure changes in cascade step outcomes as part of ADAPT. This includes measuring the types of local solutions utilized by both systems to improve the cascade.

## Limitations

The study is not without limitations. Results only present feedback of end-users from one round of dashboard development. Importantly, findings only provide insight from introduction to the dashboard and do not take into account actual end-users’ utilization of the dashboard. Understanding of individuals’ actual use of the dashboard and feedback during active use of the dashboard is needed to ensure sustainability and utility of the dashboard beyond the study period. As such, an ongoing, iterative process is needed. Despite limitations, this is the first study to examine JLS and CMHC end-user perspectives on the usability of a cross-system data dashboard.

## Conclusion

The use of data visualization to improve public health is a rapidly growing area of study and has the ability to provide objective information regarding whether systems are meeting their goals, in this case, related to substance use service connection across the youth legal and behavioral health systems. As others continue to harness the utility of administrative data and data visualization, results from the current evaluation highlight several key considerations for researchers and other stakeholders when designing and adopting the use of data dashboards, including utilizing aesthetic features to facilitate usability, clearly defining data elements and providing support to end users regarding dashboard functions, and ensuring data definitions are consistent with local terminology and salient to end users. Findings also show promise in the use of dashboards in facilitating problem-solving and decision-making among systems involved.

### Electronic supplementary material

Below is the link to the electronic supplementary material.

Supplementary Material 1

## Data Availability

No datasets were generated or analysed during the current study.
