# Supplementary material for: Early development of local data dashboards to depict the substance use care cascade for youth involved in the legal system: qualitative findings from end users
Source: BMC Health Serv Res. 2024 May 30;24:687. doi: 10.1186/s12913-024-11126-5 (PMC11140904; doi:10.1186/s12913-024-11126-5)
Supplement: Supplementary file 1 — Supplementary Material 1 [file 12913_2024_11126_MOESM1_ESM.docx]

Focus Group Guide

Background Information

- Tell me about the extent of your use of the workbook before today. Roughly how many times have you viewed your county’s data in the workbook?

Initial Usability Questions

- How would you describe the usability of the workbook? As in, what aspects of the workbook are easy to use? What aspects of the workbook are difficult to use?
- Did you think the visualizations were clear? That is, was it easy for you to extract the information that you needed from the views?
  - If there are views that you found difficult to understand, could you point them out? What was the issue with those views?
  - Did you feel the workbook provided you with all the information that you needed? Or are there missing data pieces that you would have liked to see added?
- Do you enjoy using the workbook? Why or why not?

Contextual Interview

*This section of the interview is largely unstructured; this process involves the participant sharing insights they have gathered through using the workbook or reviewing the visualizations during the interview process. The interviewee will have access to the county’s workbook via the Tableau Server and display the Tableau Workbook to the interviewer using the screen share feature on Zoom. The participant will direct the interviewer through the use of the workbook. This is intended to be an interactive process whereby the views that were related to insights gleaned are displayed. The goal is to gain an understanding of what specific features and views of the workbook are useful in gaining insights.*

- What gaps have you identified in care connection for your own county’s justice involved youth by using the workbook? OR what gaps do you notice looking at the data now?
  - Talk/walk me through how you used the workbook to gather that information.
- By looking at the workbook, what parts of the care connection process appear to be going well?
  - Talk/walk me through how you used the workbook to gather that information.
- Is there anything else you found out in the visualization that you thought was interesting/weird/insightful/etc.?
- Is there anything else about the workbook that you would like to comment on or suggest for improvement?
- Would you be interested in incorporating the use of the workbook into your work after ADAPT ends?
